# Involvement of insulin-degrading enzyme in the clearance of beta-amyloid at the blood-CSF barrier: Consequences of lead exposure

**DOI:** 10.1186/1743-8454-6-11

**Published:** 2009-09-11

**Authors:** Mamta Behl, Yanshu Zhang, Wei Zheng

**Affiliations:** 1 School of Health Sciences, Purdue University, West Lafayette, IN 47907, USA

## Abstract

**Background:**

Alzheimer's disease (AD) is characterized by the deposition of beta-amyloid (Aβ) peptides in the brain extracellular matrix, resulting in pathological changes and neurobehavioral deficits. Previous work from this laboratory demonstrated that the choroid plexus (CP) possesses the capacity to remove Aβ from the cerebrospinal fluid (CSF), and exposure to lead (Pb) compromises this function. Since metalloendopeptidase insulin-degrading enzyme (IDE), has been implicated in the metabolism of Aβ, we sought to investigate whether accumulation of Aβ following Pb exposure was due to the effect of Pb on IDE.

**Methods:**

Rats were injected with a single dose of Pb acetate or an equivalent concentration of Na-acetate; CP tissues were processed to detect the location of IDE by immunohistochemistry. For *in vitro *studies, choroidal epithelial Z310 cells were treated with Pb for 24 h in the presence or absence of a known IDE inhibitor, N-ethylmaleimide (NEM) to assess IDE enzymatic activity and subsequent metabolic clearance of Aβ. Additionally, the expression of IDE mRNA and protein were determined using real time PCR and western blots respectively.

**Results:**

Immunohistochemistry and confocal imaging revealed the presence of IDE towards the apical surface of the CP tissue with no visible alteration in either its intensity or location following Pb exposure. There was no significant difference in the expressions of either IDE mRNA or protein following Pb exposure compared to controls either in CP tissues or in Z310 cells. However, our findings revealed a significant decrease in the IDE activity following Pb exposure; this inhibition was similar to that seen in the cells treated with NEM alone. Interestingly, treatment with Pb or NEM alone significantly increased the levels of intracellular Aβ, and a greater accumulation of Aβ was seen when the cells were exposed to a combination of both.

**Conclusion:**

These data suggest that Pb exposure inhibits IDE activity but does not affect its expression in the CP. This, in turn, leads to a disrupted metabolism of Aβ resulting in its accumulation at the blood-CSF barrier.

## Background

Studies in literature have shown that more than 90% of Alzheimer's disease (AD) cases are sporadic in nature [1], suggesting the involvement of environmental triggers in addition to genetic mutations. Amongst occupational and environmental factors, exposure to toxic metal lead (Pb) has been associated with severe memory deficits and AD-like pathology as indicated by studies on Pb-exposed workers [2,3]. The deleterious effects of Pb were documented as early as in 1975, when a patient who survived severe Pb encephalopathy at 2 years of age, died of severe mental deterioration at the age of 42; neurofibrillary tangles and senile plaques, two hallmarks of AD pathology, were detected in his forebrain with significant hippocampal degeneration [4]. Other evidence linking Pb exposure and memory deficits stems from recent studies conducted on cohorts of workers who have been occupationally exposed to Pb. When their brains were examined by magnetic resonance imaging (MRI), extensive brain atrophy was seen in the cortical, hippocampal and ventricular regions that is typical of AD patients. The brain atrophy and neurobehavioral deficits in these workers appeared to persist several years post Pb exposure and correlate with a high tibia Pb concentration, a marker often used to estimate cumulative Pb exposure [2,3,5-7]. In animal models, developmental exposure to Pb has been associated with an early transient and delayed over-expression of amyloid precursor protein (APP) and its amyloidogenic product, β-amyloid (Aβ) [8]. These studies and others [9,10], provide evidence to support a linkage between Pb exposure and the pathogenesis of AD.

One of the target regions in the brain where Pb has shown to accumulate substantially is the choroid plexus (CP), a tissue lining the brain ventricles and separating the blood from the CSF. This finding is supported by evidence from an autopsy study which showed an age-related accumulation of Pb in the CP of the brains of 51 human subjects who had lived in New York City [11]. This observation was later confirmed by another independent human autopsy study revealing a 100-fold increase of Pb in the CP compared with the brain cortex [12]. These findings were corroborated by studies in rodents demonstrating a dose-dependent and time-related accumulation of Pb in the CP at concentrations 57 and 70 fold greater than the brain cortex and CSF, respectively [13,14].

Recent studies from this laboratory have demonstrated that Pb accumulation in the CP results in a marked increase of Aβ within the tissue [15]. Since the CP plays a significant role in Aβ transport and clearance from the CSF [16], the objective of this study was to investigate the mechanism by which Pb interferes with the metabolic clearance of Aβ, specifically its degradation by insulin-degrading enzyme (IDE).

IDE, a 110-KD zinc metallopeptidase, is known to participate extensively in the clearance of insulin, glucagon and Aβ [17-21]. The common physicochemical characteristics shared by IDE substrates include hydrophobicity and the substrate's ability to aggregate readily to form fibrils [18,22,23]. IDE is known to be present in the cytosol, peroxisomes as well as in the mitochondria of neuronal and non-neuronal cells in the brain [24-26]. It has also been detected in the human CSF of both normal and Alzheimer subjects [20,21,27]. Noticeably, a decrease in IDE has been associated with impaired neuronal regulation of Aβ as well as deficits in spatial memory in both rats and AD patients [28-31]. Other studies involving genetic deletions of IDE in mice have shown a significant elevation in both, brain Aβ and plasma insulin [25,32]. Interestingly, an up-regulation of IDE in neurons has been shown to prevent against AD-like pathology in transgenic mice which over-express APP [25,33]. Recently, IDE has been identified in the CP by our group, although its exact role in Aβ metabolic clearance remains unclear [34].

Considering the substantial accumulation of Pb and the abundant presence of IDE in the CP, there was sound reason to hypothesize that accumulation of Pb may alter the enzymatic activity or expression levels of IDE in the CP, leading to alterations in Aβ metabolism at the blood-CSF barrier (BCB). Thus, the purpose of this study was to (1) identify the subcellular location of IDE in the CP, (2) determine whether Pb exposure *in vivo *affected the subcellular distribution of IDE, (3) investigate whether Pb exposure altered the IDE activity and/or the expression of mRNA or protein levels in the CP, and (4) determine whether altered IDE function led to the abnormal accumulation of Aβ in the BCB. The outcomes of this study will establish the role of IDE in the metabolic clearance of Aβ at the BCB and illustrate a mechanism by which Pb alters Aβ homeostasis in the CSF, potentially contributing to the etiology of AD.

## Methods

### Materials

Chemicals and assay kits were purchased as follows: ELISA kit and ultra purified Aβ (1-40) (#KHB-3841 and #03-136, Biosource, Carlsbad, USA); mouse anti -IDE antibody (#MMS 282R, Covance Inc, Princeton, USA); IDE activity kit (# CBA079, Calbiochem Gibbstown, USA); Alexa-labeled secondary anti- mouse antibody (Molecular Probes, Eugene, USA); enhanced chemiluminescene reagent (ECL) and ECL films (Amersham Biosciences, Piscataway, USA); Dulbecco's modified essential medium (DMEM), fetal bovine serum (FBS), penicillin, streptomycin, and gentamycin (Gibco, Grand Island, USA); PCR buffer, dNTP, Oligo dT and MuLV reverse transcriptase (Applied Biosystems, Foster City, USA); the ABsolute QPCR SYBR green Mix kit (ABgene, Rochester, USA); the primers for real time RT-PCR (Integrated DNA Technology Inc., Coralville, USA); β-actin, 2-mercaptoethanol, phenylmethylsulfonyl fluoride (PMSF), polyacrylamide, tetramethyl-ethylenediamine (TEMED), n-ethylmaeimide (NEM) and all other chemicals (Sigma Chemicals, St. Louis, USA). All reagents were of analytical grade, HPLC grade or the best available pharmaceutical grade.

### Animals and treatment

Male Sprague-Dawley rats were purchased from Harlan Laboratories (Indianapolis, USA) and were 8-9 weeks old (250-300 g) at the time they were used. The animals were housed in a temperature-controlled, 12 h:12 h light/dark room, and were allowed free access to tap water and food. Rats received an i.p. injection of 50 mg/kg Pb acetate (i.e., 27 mg Pb/kg) or an equivalent molar concentration of Na-acetate (i.e., 15 mg acetate/kg) as control. Twenty-four h post injection, the rats were anesthetized with an injection of ketamine/xylazine (75:10 mg/mL, 1 mL/kg body weight) and euthanized by exsanguination to remove excess blood. The lateral ventricle CP was then isolated for further experimentation. Animal protocols pertinent to this study were approved by the Purdue University Animal Care and Use Committee.

### Culture of choroidal epithelial Z310 cells

The methods and characteristics of immortalized rat choroidal epithelial Z310 cells have been described previously [35]. Briefly, cells were maintained in DMEM (high glucose) medium supplemented with 10% FBS, 100 U/mL penicillin, 100 μg/mL streptomycin, and 40 μg/mL of gentamycin in a humidified incubator with 95% air-5% CO_2 _at 37°C and were passaged twice a week. The cells were seeded at a density of 1 × 10^6 ^in a 100-mm culture dish. Twenty-four h after initial seeding, the cells were treated with 0 or 10 μM Pb for 24 or 48 h, and the following studies were performed.

### Immunohistochemistry

Immunohistochemistry was performed on both rat CP tissues and the Z310 cells following Pb exposure. For fresh tissue studies, the CPs were isolated from the rat following the exposure condition described above and transferred to a 35-mm glass bottomed dish containing PBS. For *in vitro *studies, Z310 cells were seeded on a 35-mm dish, and treated with 10 μM Pb for 24 or 48 h. At the end of Pb treatment, the culture medium was removed and replaced with 0.5-1 mL PBS. The tissues or cells were washed with PBS three times, fixed with 3% paraformaldehyde/0.25% glutaraldehyde in PBS for 10 min, and permeabilized with 0.5% Triton X-100 for 30 min at room temperature, followed by 3-5 washes of PBS. After blocking with 1% bovine serum albumin (BSA) in PBS for 30 min at room temperature, the tissues or cells were double-immunostained with mouse anti-IDE (1:500) in 1% BSA overnight at 4°C, washed the next day with PBS in 1% BSA, and then incubated with goat anti- mouse Alexa-488 conjugated secondary antibody (1:7500) in 1% BSA at 37°C for 60 min. After further washing in PBS with 1% BSA, the tissues or cells were observed using an inverted confocal fluorescent microscope. Negative controls were treated similarly except that they were not exposed to any of the primary antibodies.

### Confocal immunofluorescence microscopy

To acquire images, the dish containing the CP specimen was mounted on the stage of an Olympus, FV1000 inverted confocal laser-scanning microscope and viewed through a 40× water-immersion objective (numeric aperture = 1.2), with a 488-nm laser line for excitation (Ar-ion laser). Low laser intensity was used to avoid photo bleaching. The CP was examined under a reduced transmitted-light illumination. An area containing undamaged epithelium with underlying vasculature was selected. For each image acquired (512 × 512 × 8 bits, 4 frames averaged), four areas of the specimen were selected for image collection. Data reported, unless otherwise stated, are representative of 3-4 replicate experiments.

### Assay of IDE enzymatic activity

To screen for a suitable NEM concentration at which cell death was minimal, Z310 cells were seeded at a density of 1 × 10^6 ^in 100-mm plates for 24 h; they were then incubated with 0, 5, 10, 25 or 50 μM of NEM overnight for 16 h and the cell viability was determined using the methylthiazolyldiphenyl-tetrazolium bromide (MTT) assay. Based on our results and previously published data [21], a concentration of 10 μM NEM was selected as an appropriate concentration for the IDE activity experiments described below.

To assess the IDE activity, the cells were seeded at a density of 1 × 10^6^and treated with Pb for 24 or 48 h in the presence or absence of NEM. The four treatment conditions were as follows: Group 1 served as an untreated control (Pb-/NEM-), group 2 was treated with 10 μM Pb for 24 h in the absence of NEM (Pb+/NEM-), group 3 was treated with only 10 μM NEM for 16 h in the absence of Pb (Pb-/NEM+), and group 4 with 10 μM NEM for 16 h followed by 10 μM Pb for 24 h (Pb+/NEM+).

The principle of the assay is based on measuring the IDE activity using a fluorescence resonance energy transfer (FRET) substrate which is cleaved by IDE to release the fluorophore from the quenching molecule, resulting in an increase in fluorescence. The activity was determined as per manufacturer's protocol (Calbiochem, catalog # CBA079). Briefly, Z310 cells were lysed with RIPA buffer and centrifuged at 10,000 rpm for 10 min and the supernatant was used for analysis. Total protein (80 μg/well) was loaded into in a 96-well plate, which contained an affinity-purified polyclonal antibody that recognizes human, mouse, and rat insulysin; the antibody was immobilized on the plate in order to capture the IDE enzyme. Following 1 h incubation, the plate was washed with sample buffer, and an aliquot (100 μL) of substrate was added and incubated for 2-4 h at 37°C in the dark. The fluorescence was measured using an excitation wavelength of 320 nm and an emission wavelength of 405 nm. The results are reported as IDE fluorescence intensity in arbitrary units (a.u.)/mg of total proteins in the cells.

### Quantification of Aβ_1-40 _accumulation in Z310 cells following Pb exposure by ELISA

Cells were grown on 24 well plates at a density of ~4 × 10^4 ^cells/well and treated as the four groups described above. The cells were washed with PBS and incubated with 200 μL of 2 μM ultrapure Aβ_1-40 _solution in serum free medium for 1 h. The medium was then removed, cells washed 3 times with PBS, and fresh serum-free medium added for an additional hour to allow for metabolic clearance of Aβ by IDE. The cells were then washed with PBS, scraped and lysed using 60 μL of RIPA buffer. The intracellular Aβ was determined using a colorimetric ELISA kit (Invitrogen KHB3481). All data reported from the ELISA were normalized to the total amount of protein in the cells as determined by the Bradford assay.

### Quantification of IDE mRNA expression by real-time (RT)-PCR

The transcription of the gene encoding IDE was quantified using RT-PCR as described in [36]. Briefly, the total RNA was isolated from Z310 cells or plexus tissue using TRIzol reagent, following the manufacturer's instructions. An aliquot of RNA (1 μg) was reverse-transcribed with MuLV reverse-transcriptase and oligo dT primers. The forward and reverse primers for target genes were designed using Primer Express 3.0 software. The ABsolute QPCR SYBR green (Mix kit, ABgene) was used for RT-PCR analyses. The amplification was carried out in the MX 3000P real-time PCR System (Stratagene, La Jolla, USA). Amplification conditions were 15 min at 95°C, followed by 40 cycles of 30 s at 95°C, 1 min at 55°C and 30 s at 72°C. A dissociation curve was used to verify that the majority of fluorescence detected could be attributed to the labeling of specific PCR products, and to verify the absence of primer-dimers and sample contamination. All RT-PCR reactions were done in triplicate. Primer sequences for rat IDE used for real-time RT-PCR were: forward primer 5' GGTTGGAGAGTTCCCCTCTCA-3' and a reverse primer 5' AGGCCGCGCTTGAATTC-3' (Genbank Accession No NM_013159) and rat glyceraldehydes-3-phosphate dehydrogenase (GAPDH) used as an internal control, had a forward primer 5'-CCT GGA GAA ACC TGC CAA GTA T-3' and a reverse primer 5'-AGC CCA GGA TGC CCT TTA GT-3' (Genbank Accession No. NM_017008).

### Quantification of IDE protein expression by western blot

The plexus tissues or Z310 cells were homogenized (1:10, wt/vol) on ice in a buffer containing 20 mM Tris (pH 7.5), 5 mM EGTA, 1% TritonX-100, 0.1% SDS, 50 μM phenylmethylsulphonylfluoride (PMSF), 15 mM 2-mercaptoethanol and a protease inhibitor cocktail containing 500 μM 4-(2-Aminoethyl) benzenesulfonyl fluoride hydrochloride (AEBSF), 150 nM aprotinin, 1 μM E-64, 0.5 mM EDTA, 1 μM leupeptin (Calbiochem). Samples were sonicated using a Model 500 Sonic Dismembrator (Fisher Scientific) at duty cycle 20% and output 4-6 for 30 pulses. Following centrifugation at 10,000 g at 4°C for 10 min, aliquots of supernatants were assayed for protein concentrations by the Bradford method. A volume of protein extract (40 μg of protein) was mixed with an equal volume of 2× sample buffer (0.35 M Tris-Cl, 10% SDS, 30% glycerol, 0.6 M DTT, and 0.012% bromophenol blue), loaded onto a 10% SDS-polyacrylamide gel, electrophoresed, and then transferred to a PVDF membrane. The membrane was blocked with 5% dry milk in TBST (tris-buffered saline) at room temperature for 1 h and immunoblotted with an antibody directly against IDE (1:250). The membrane was stained with a horse-radish peroxidase (HRP)-conjugated goat anti-mouse IgG antibody (1:5000) at room temperature for 1 h and developed using ECL reagent. The exposure time varied from 30 sec to several min depending on the signal strength. β-actin (42 kD) (1:2000) was used as a loading control; the corresponding secondary antibody (1:2000) for β-actin was HRP-conjugated goat anti-mouse IgG. All band intensities were quantified using Scion Image software (Frederick, USA).

### Determination of Pb-induced cellular toxicity in Z310 cells

To determine the Pb concentration at which it altered Aβ metabolism in the CP in the absence of nonspecific cytotoxicity, three general cytotoxicity assays were conducted including the MTT cell viability assay, cell membrane permeability assessment (lactate dehydrogenase or LDH assay), and cellular oxidative stress estimation (superoxide dismutase or SOD assay). The MTT assay was performed by culturing Z310 cells at a density of 15,000 cells/well in a 96 well plate for 2-3 days until they reached 70% confluence. The medium was then replaced with fresh medium containing different concentrations of Pb as Pb-acetate (0, 1, 5, 10, 50, 100 μM). The cells were incubated for an additional 24 h, followed by adding an aliquot of MTT stock solution (2 mg/mL in PBS) to each well. The absorbance of the converted dye was measured at a wavelength of 570 nm. To determine the LDH activity, Z310 cells were treated as described above and LDH released into the culture medium was determined using the assay kit according to manufacturer's protocol. Finally, to determine oxidative stress, the cells were exposed to Pb (10 μM) for 24 h and SOD activity was determined according to the instructions in the assay kit.

### Statistical analysis

Statistical analyses were carried out by a one-way ANOVA with *post hoc *comparisons using the Dunnett's test or paired t-tests (Kaleidagraph 3.6). All data are expressed as mean ± SD. Differences between two means were considered significant when the *p *value was equal or less than 0.05.

## Results

### Subcellular localization of IDE in choroid plexus tissues, choroidal epithelial Z310 cells and the effect of Pb

**Figure 1:**
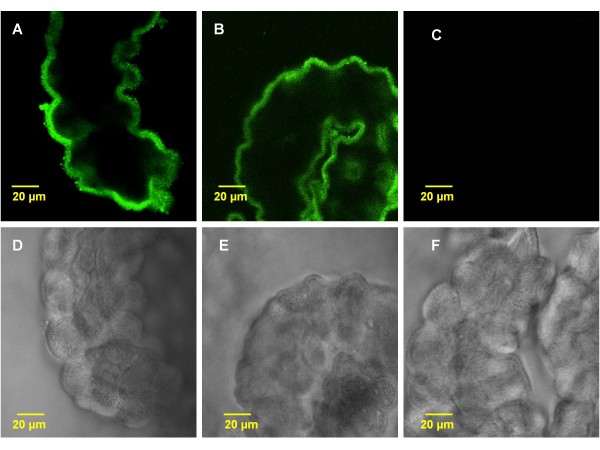
**Confocal immunofluorescence images showing intracellular location of IDE in rat choroid plexus tissue following *in vivo *acute Pb exposure**. A: The plexus tissues from a control rat. The IDE signals were present near the apical membrane of the epithelia, facing the CSF. B: Rats received a single ip injection of 27 mg Pb/kg. Twenty-four hours post exposure, there was no apparent sub-cellular re-localization of IDE following Pb exposure. C: Negative control without primary antibody. D, E, F: Transmission microscopy images. Note: The tissues in rats exposed to Pb (E) did not exhibit morphological alterations compared with controls (D). The images are representative of experiments run in triplicate.

Immunostaining of normal rat lateral ventricle CP tissues revealed a distinct staining of IDE in the choroidal epithelia (Fig. 1). The IDE staining was primarily present in the cytosol towards the apical membrane including microvilli. There was very little staining in the cytosol towards the basement membrane facing the blood side (Fig. 1A). Acute *in vivo *exposure to Pb did not alter the subcellular localization of IDE, nor did it seem to affect the intensity of IDE staining (Fig. 1B). As expected, there was no staining in the negative control, which was the group treated in the absence of primary antibody (Fig. 1C). Noticeably, the corresponding transmission images revealed a normal morphology of the CP tissues following acute *in vivo *Pb exposure (Fig. 1D, E).

**Figure 2:**
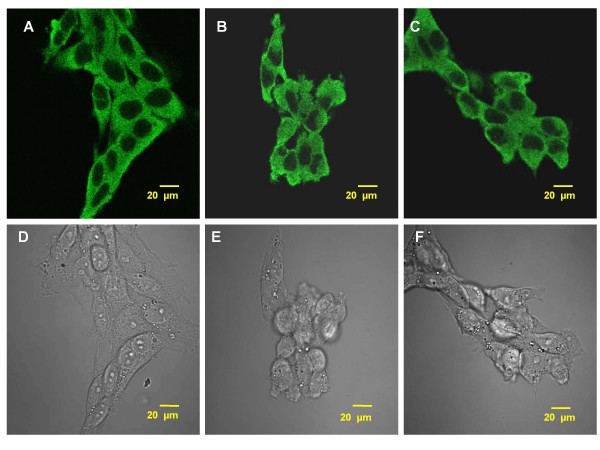
**Confocal immunofluorescence images showing intracellular expression of IDE in rat choroidal epithelial Z310 cells following Pb (10 μM) exposure**. A: Z310 cells from untreated, control group. B: Z310 cells exposed to Pb for 24 h. C: Z310 cells exposed to Pb for 48 h. No apparent subcellular re-localization of IDE was observed following Pb exposure in any of these groups. Transmission microscopy images indicate that the Pb treated cells (E, F) did not exhibit any morphological alterations compared to controls (D). The images are representative of experiments run in triplicate.

The IDE staining was also detected in choroidal epithelial Z310 cells with fluorescent signals evenly distributed in the cytosol (Fig. 2A). Since these cells lack polarity, no particular directional subcellular distribution of IDE was expected. Treatment with Pb (10 μM) for 24 or 48h did not change either the localization or intensity of IDE staining in these cells (Fig. 2B, C). Transmission images revealed a normal morphology of Z310 cells (Fig. 2D, E, F).

### Acute Pb exposure decreases IDE activity in choroidal epithelial Z310 cells

Prior to analyzing the IDE activity, it was necessary to ensure that the concentrations of Pb used in this study did not cause any direct cytotoxicity to the cells. The MTT, LDH and SOD assays were conducted for this purpose. The MTT assay with Pb concentration ranging between 0-150 μM revealed 95% viability at 10 μM Pb compared to controls. The LDH assays revealed that a concentration of Pb at or below 10 μM had no significant effect on LDH release. Furthermore, results from the SOD assay did not show any significant oxidative stress when the cells were exposed to Pb at 10 μM. According to these findings, together with previously published data from this group [37], a concentration of 10 μM Pb was selected for the following studies.

**Figure 3:**
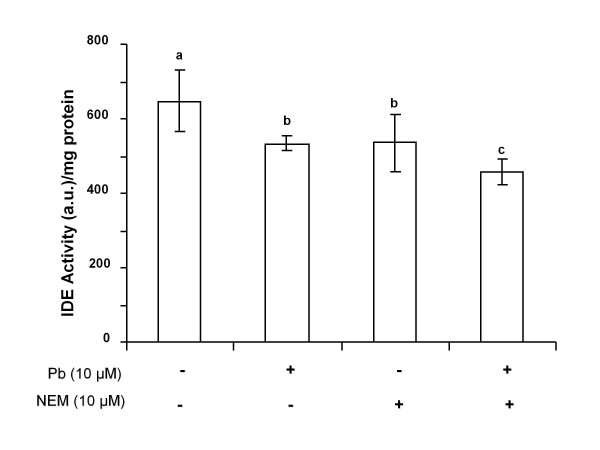
**IDE activity following Pb exposure (10 μM, 24 h) in rat choroidal epithelial Z310 cells in the presence (+) or absence (-) of IDE inhibitor, NEM (10 μM)**. Data represent mean ± SD, n = 6-8 wells per group. Bars with different superscripts are significantly different from one another, *p *< 0.05.

For the IDE activity study, NEM, an effective IDE inhibitor, was used as a positive control in the presence or absence of Pb exposure. Cells exposed to Pb for 24 h in the absence of NEM (Pb+, NEM-) showed a significant decrease of 17.6% in their IDE activity (*p *< 0.05) compared to untreated controls (Pb-, NEM-) (Fig. 3). Treatment with NEM alone (10 μM) (Pb-, NEM +) resulted in a 17.5% decrease in the IDE activity (*p *< 0.05) and was comparable to the group treated with Pb in the absence of the inhibitor (Pb+, NEM-). When the cells were exposed to a combination of Pb and NEM (NEM+, Pb+), the IDE activity was reduced by a significant 29.3% (*p *< 0.001) relative to untreated controls (NEM-, Pb-), by 11.7% (*p *< 0.05) relative to the cells exposed to Pb alone (Pb+, NEM-), and by 11.8% (*p *< 0.05) relative to the NEM alone group (Pb-, NEM +). NEM did not cause any significant cell death at the concentrations used, as determined by the MTT assay. These results suggested that exposure to Pb (10 μM) results in a decrease in IDE activity; the effect of Pb on the enzymatic activity of IDE was exacerbated in the presence of IDE inhibitor, NEM.

### Inhibition of IDE activity by Pb increases intracellular Aβ accumulation

**Figure 4:**
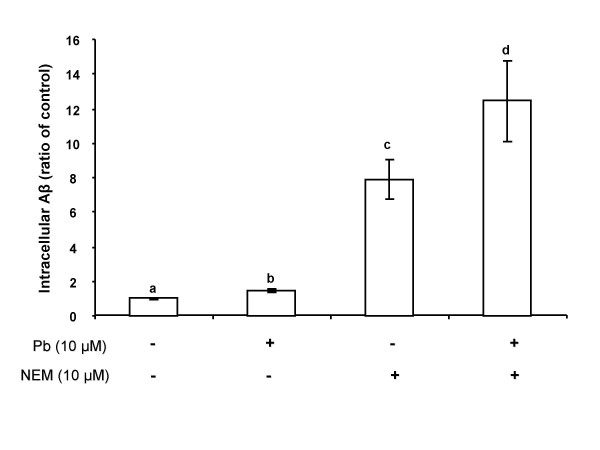
**Quantification of intracellular Aβ levels by ELISA in Z310 cells following Pb (10 μM, 24 h) exposure in the presence (+) or absence (-) of NEM (10 μM)**. Data represent mean ± SD, n = 6-8 wells per group. Bars with different superscripts are significantly different from one another, *p *< 0.05.

To determine if the inhibition of IDE activity led to cellular accumulation of Aβ, Z310 cells were incubated with Aβ (2 μM) following treatment with Pb and/or NEM. Analysis using a one way ANOVA revealed an overall significant difference between the groups (*p *< 0.01, Fig. 4). The cells treated with Pb alone (Pb+, NEM-) showed a relatively small (1.5 fold), yet statistically significant increase in intracellular Aβ levels compared to untreated controls (*p *< 0.05). Treatment with NEM alone (Pb-, NEM+) resulted in a 7 fold increase in intracellular Aβ concentrations compared to untreated controls (*p *< 0.01).

When both Pb and NEM (Pb+, NEM+) were added to the culture system, the intracellular Aβ levels were increased by nearly 12 fold (*p *< 0.01). This finding supports a synergistic effect between Pb and NEM; both may possibly alter the IDE activity and subsequently decrease Aβ metabolism in the CP.

### Pb exposure did not alter the expression of IDE mRNA and protein in CP tissues or choroidal Z310 cells

**Figure 5:**
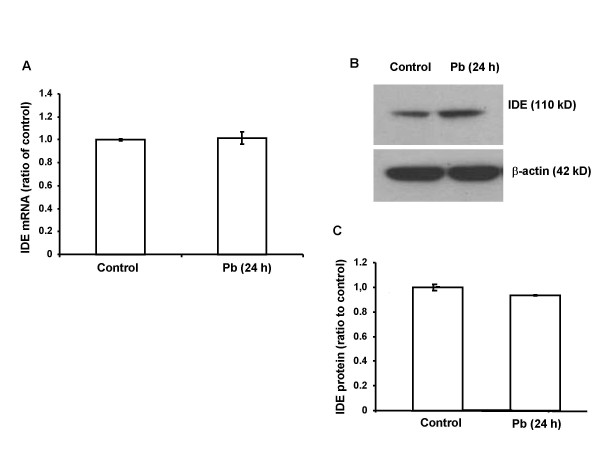
**IDE mRNA and protein expression following Pb exposure in rat CP tissue**. Rats received ip injection of either Na acetate (control) or Pb acetate (27 mg Pb/kg) and tissues were analyzed 24 h after Pb exposure. A: IDE mRNA expression. No significant difference was observed between Pb-exposed and control groups as indicated by the ratio of IDE/GAPDH. B: Representative western blots of IDE protein expression in controls and Pb-treated rats. C: IDE expression estimated from the corresponding band densities in (B) and normalized to those of β-actin indicating. No significant difference was observed in IDE protein expression between Pb-treated and control rats. Data represent mean ± SD, n = 4. The data are representative of triplicate experiments.

**Figure 6:**
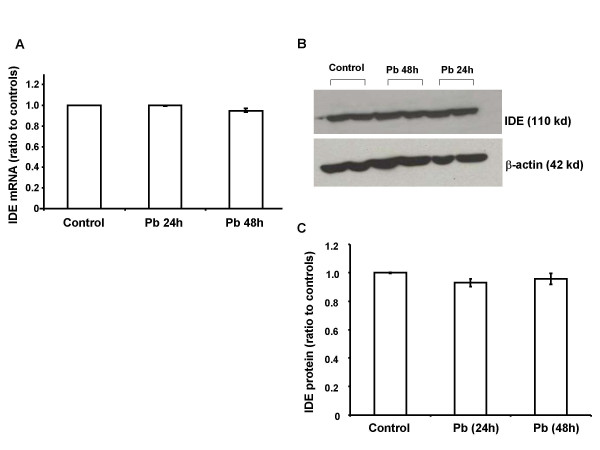
**IDE mRNA and protein expression in Z310 cells following Pb exposure**. Z310 cells were treated with 10 μM Pb for 24 h or 48 h. A: IDE mRNA expression. No significant difference was observed in the mRNA expression between the Pb-exposed and control groups. B: Representative western blots of IDE protein expression in controls and cells treated with Pb (10 μM) for 24 and 48 h. C: IDE expression estimated from the corresponding band densities (B) and normalized to those of β-actin. No significant difference in IDE protein expression was found between Pb-treated and control cells. Data represent mean ± SD, n = 3. The data are representative of triplicate experiments.

The effect of Pb on IDE function could also be due to the altered expression of IDE. To test this hypothesis, rats were exposed to Pb by a single injection of 27 mg Pb/kg i.p. for 24 h. No significant difference was observed either in the IDE mRNA expression level as quantified by real time RT-PCR (Fig. 5A), or in the protein expression level as determined by western blot analysis (Fig. 5B, C). In addition, there was an absence of a significant alteration in mRNA (Fig. 6A) and protein (Fig. 6B, C) in choroidal Z310 cells treated with 10 μM Pb for 24 or 48 h.

## Discussion

Our data clearly demonstrate that (i) IDE is located towards the apical membrane in CP tissues and the distribution does not appear to be altered by Pb exposure; (ii) Pb exposure results in a significant decrease in IDE activity; (iii) the decrease in IDE activity by Pb may lead to an accumulation of Aβ in the CP possibly due to a reduced metabolic clearance at the BCB; and (iv) Pb exposure does not alter the mRNA or protein expression of IDE.

The observation that the subcellular location of IDE was concentrated towards the apical membrane has significant implications. Earlier studies in our laboratory have demonstrated that the direction of Aβ transport by the CP is predominantly from the CSF to the blood [16]. The presence of IDE immediately beneath the apical membrane would allow this enzyme to effectively metabolize the Aβ molecules entering from the CSF. Hence, the immediate breakdown of Aβ by IDE may facilitate the ability of the CP to continuously attract extracellular Aβ; this may also explain the large capacity of the CP to take up Aβ from the CSF [16].

Recent studies in our laboratory have shown that exposure to Pb results in the accumulation of intracellular Aβ [15]; the study employed the same Pb exposure dose regimen as used in the current experiment. Under such a dose regimen, the Pb concentrations in blood and CSF reach 400 μg/dL and 32 μg/dL, respectively; the Pb concentration in the CP is about 22 μg/g of tissue, nearly 57 fold greater than Pb in brain cortex [13]. Recently published data by this group also demonstrate that incubating Z310 cells with 1-10 μM of Pb (about 20.7-207 μg/dL in the culture medium) produced a dose-time dependent increase in cellular accumulation of Aβ while not causing significant cell damage (i.e., normal cell viability, normal LDH and normal SOD) [15]. This basic finding could be explained at least in part by Pb facilitating the uptake of Aβ by the CP and/or Pb inhibiting Aβ breakdown in the CP. The data in the current study indicated that Pb may directly act on IDE by inhibiting its activity rather than altering its gene or protein expression.

Results from this study indicated that while Pb exposure caused a 1.5 fold increase in Aβ, the group treated with NEM alone caused a 7 fold increase in Aβ. This may be, in part, due to a non-IDE-related effect of NEM, since NEM action involves a general covalent binding to cysteine residues. One strategy for future studies would be to knock-down IDE using short interference RNA and to more specifically determine the contribution of IDE. Another approach would be to up-regulate IDE in these cells and analyze Aβ accumulation in the presence or absence of Pb exposure. We would also like to point out that due to a small tissue mass, we were unable to assess the IDE activity in the CP tissue following *in vivo *Pb exposure. As a future direction, it would be of interest to know how our *in vitro *studies correlate with *in vivo *IDE activity in the CP.

The current study raises several interesting questions. By what potential mechanism does Pb exert its effects on IDE? Extensive literature suggests that Pb is capable of binding to Zn finger proteins and subsequently altering their function, specifically at the sp1 binding domain [38-40]. IDE activity is shown to be metal or thiol-dependent [19,41] and structurally comprises a Zn binding motif, as well as an inverted metalloprotease motif named HxxEH [24]. These, along with several cysteine-histidine clusters, allow the enzyme to bind to substrates such as Aβ and assist in its cleavage. At the same time, IDE could potentially interact with DNA to regulate transcriptional events [42]. Pb, being a divalent metal like Zn may compete with and replace Zn in the Zn finger binding domain. At the transcriptional regulatory level, this binding can alter the upstream signaling pathways that are important to cell function [43-45]. Since the structure of IDE contains a Zn motif, we speculate that Pb may directly bind to the Zn finger binding domain and inhibit IDE activity. However, this is a preliminary speculation and further studies are warranted to explore this mechanism.

Upstream of binding to the Zn finger, Pb might also influence the protein kinase C (PKC) pathway. Studies in literature suggest that IDE activity is decreased by an energy-dependent ATP mechanism [46]. PKC is known to phosphorylate amino acids by ATP-driven reactions and has been implicated in Pb toxicity in both animals and in humans [47-51]. Specifically, findings reveal that exposure to Pb results in PKC- mediated impairments in hippocampal development, the brain area that is primarily affected in AD [40]. Since Pb has shown to activate several isoforms of PKC in the CP [52], it would be of interest to explore whether and how activation of PKC may relate to alterations in IDE activity.

Another question is how much of a role does IDE play in Pb-mediated accumulation of Aα? We should point out that this study does not imply that IDE is the single most important factor in Aβ regulation at the BCB. Other mechanisms, particularly receptor-mediated Aβ uptake and clearance in the CP including the involvement of Aα transporters such as low density lipoprotein receptor protein 1 (LRP1), receptor for advanced glycosylation end products (RAGE) and p-glycoprotein must also be considered.

Finally, what is the consequence of Aβ accumulation in the CP following Pb exposure? A build up of Aβ in the CP could either be a result of overproduction of Aβ in the CNS under the interference of Pb, or maybe due to a compromised function of the CP in clearing Aβ from the CSF. Data from the current study indicate that Pb may alter the Aβ breakdown mechanism at a critical site in the brain that is often overlooked. A disruption in Aβ clearance, in turn, might lead to elevated Aβ accumulation in the brain's internal milieu. Although this study provides some evidence for an IDE-mediated metabolic clearance, further studies are warranted to explore this mechanism in-depth.

## Conclusion

In summary, our results provide evidence that Pb exposure increases Aβ accumulation at the BCB at least in part, by altering the activity of IDE and thereby potentially decreasing the metabolic clearance of Aβ. In addition, we provide evidence that IDE in the CP is localized towards the apical membrane, which is consistent with the large capacity of the CP to take up Aβ from the CSF. Finally, our study suggests that Pb is a potential environmental trigger in the dysregulation of Aβ homeostasis, and may subsequently contribute to the etiology of AD.

## Abbreviations

Aβ: beta-amyloid; AD: Alzheimer's disease; BCB: blood-CSF barrier; CP: choroid plexus; CSF: cerebrospinal fluid; ELISA: enzyme-linked immunosorbent assay; IDE: insulin degrading enzyme; LDH: lactate dehydrogenase; MRI: magnetic resonance imaging; MTT: methylthiazolyldiphenyl-tetrazolium bromide; NEM: n-ethylmaleimide; Pb: lead; PKC: protein kinase C; RT-PCR: reverse transcription polymerase chain reaction; SOD: superoxide dismutase.

## Competing interests

The authors declare that they have no competing interests.

## Authors' contributions

MB participated in designing the study, performing the experiments, analyzing and interpreting the data and drafting the manuscript. YZ participated in designing the study, assisted in carrying out the experiments and analyzing the data. WZ (senior author) participated in designing the study, interpreting the data and revising the manuscript. All authors have read and approved the final manuscript.
